# Accurate Mass GC/LC-Quadrupole Time of Flight Mass Spectrometry Analysis of Fatty Acids and Triacylglycerols of Spicy Fruits from the *Apiaceae* Family

**DOI:** 10.3390/molecules201219779

**Published:** 2015-12-02

**Authors:** Thao Nguyen, Mario Aparicio, Mahmoud A. Saleh

**Affiliations:** 1 Department of Chemistry, Texas Southern University, Houston, TX 77004, USA; ltt_t@yahoo.com; 2 Agilent Technologies, Inc., 3750 Brookside Parkway, Suite 100, Alpharetta, GA 30022, USA; mario.aparicio@agilent.com

**Keywords:** seed oil, petroselinic acid, regiospecific, lipids, QTOF

## Abstract

The triacylglycerol (TAG) structure and the regio-stereospecific distribution of fatty acids (FA) of seed oils from most of the *Apiaceae* family are not well documented. The TAG structure ultimately determines the final physical properties of the oils and the position of FAs in the TAG molecule affects the digestion; absorption and metabolism; and physical and technological properties of TAGs. Fixed oils from the fruits of dill (*Anethum graveolens*), caraway (*Carum carvi*), cumin (*Cuminum cyminum*), coriander (*Coriandrum sativum*), anise (*Pimpinella anisum*), carrot (*Daucus carota*), celery (*Apium graveolens*), fennel (*Foeniculum vulgare*), and Khella (*Ammi visnaga*), all from the *Apiaceae* family, were extracted at room temperature in chloroform/methanol (2:1 *v*/*v*) using percolators. Crude lipids were fractionated by solid phase extraction to separate neutral triacylglycerols (TAGs) from other lipids components. Neutral TAGs were subjected to transesterification process to convert them to their corresponding fatty acids methyl esters (FAMES) using 1% boron trifluoride (BF_3_) in methanol. FAMES were analyzed by gas chromatography-quadrupole time of flight (GC-QTOF) mass spectrometry. Triglycerides were analyzed using high performance liquid chromatography-quadrupole time of flight (LC-QTOF) mass spectrometry. Petroselinic acid was the major fatty acid in all samples ranging from 57% of the total fatty acids in caraway up to 82% in fennel. All samples contained palmitic (16:0), palmitoleic (C16:1n-9), stearic (C18:0), petroselinic (C18:1n-12), linoleic (C18:2n-6), linolinic (18:3n-3), and arachidic (C20:0) acids. TAG were analyzed using LC-QTOF for accurate mass identification and mass spectrometry/mass spectrometry (MS/MS) techniques for regiospesific elucidation of the identified TAGs. Five major TAGs were detected in all samples but with different relative concentrations in all of the tested samples. Several other TAGs were detected as minor components and were present in some samples and absence in the others. Regiospecific analysis showed a non-random fatty acids distribution. Petroselinic acid was predominantly located at the sn-1 and sn-3 positions.

## 1. Introduction

The botanical *Apiaceae* family also known as *Umbelliferae* or the parsley family is one of the major families for culinary herbs and root crops comprising up to 400 genera of plants distributed throughout a wide variety of habitats in the temperate climate regions of the world [1]. Plants are widely used as vegetables, food spices, herbal folk remedies and as ornamentals [2]. Many seed oils of the family are rich in petroselinic acid (6*Z*-octadec-6-enoic acid) also known as 18:1n-12, which represent an interesting oleochemical for the food, cosmetics, and pharmaceutical industries [3,4]. Most medicinal and aromatic plants are often used as spices, vegetables or drugs owing to the presence of useful secondary metabolites. Some of them are known for their high level of polyunsaturated fatty acid in seed oil such as the fruits of the *Apiaceae* family [5,6].

The triacylglycerol (TAG) structure and the regio- and stereospecific distribution of fatty acids (FA) of seed oils from most of the *Apiaceae* family are not well documented. FA analysis can be used to evaluate the composition, stability and nutritional quality of fats and oils, but not their functional properties; however, the TAG structure ultimately determines the final physical properties of the oils. The position of FAs in the TAG molecule affects the digestion, absorption and metabolism, and physical and technological properties of TAGs, as it is known that gastric and pancreatic lipases are regioselective. Thus, the objectives of the present work were to use accurate mass spectrometry to identify the structure and regiostereochemistry of TAG species present in selected common seed oils of the *Apiaceae* family.

## 2. Results and Discussion

### 2.1. Fatty Acids Composition

Oil yield for extracted fruit ranged from 10 to 23 g/100 g of fruits with cumin giving the highest yield of oil and celery being the least, as shown in Table 1. Fatty acids compositions of the purified neutral triglycerides were analyzed in the form of methyl esters using accurate mass (GC-QTOF) mass spectrometry. Relative percentage composition of fatty acids methyl esters (FAMES) in each oil is shown in Table 1. FAMES were identified based on their retention times and their accurate mass data. Their electron ionization fragmentation and mass spectral data were also searched using Wiley10NIST mass spectral database. Petroselinic acid (6*Z*-octadecenoic acid) is a characteristic fatty acid of the *Apiaceae* family, and was found to be the major fatty acid in all of the tested samples as shown in Table 1. This acid is interesting because of its antimicrobial activity [7,8] and, because its oxidation gives lauric acid (C12:0), a very important fatty acid used in the soap, cosmetic, medical and perfume industries [9,10,11,12]. Palmitic acid (C16:0) was found in all samples with a concentration ranging from 4%–5%, except celery oil exceeding 8% of the total. Two isomers of palmitoleic acid (C16:1), the first, 9*Z*-hexadec-9-enoic acid, was found in all samples at concentration ranging from 0.3% to 0.9% of the total fatty acids composition, the second isomers was found to be 7*Z*-hexadec-9-enoic acid and was found in all samples as a minor component but was not present in dill oil. Stearic acid (C18:0) was found in all samples at a level of 1% to 2% but was not detected in the oil of cumin and coriander. Petroselinic acid (6*Z*-octadec-6-enoic acid) was the major fatty acids in all of the samples with a concentration of up to 80% of the total fatty acids in the oil of dill, coriander, carrot and fennel, with caraway oil having the least percentage in the group (58%). Oleic acid 18:1 was absent in all samples except the oils of caraway, cumin and anise where they contain less than 2%. Linolenic acid 18:2 was the second most abundance fatty acids in all samples with concentration ranging from 10% in Dill oil to as high as 31% in caraway oil. With regard to the overall yield of seed oil, cumin showed the highest yield (23.4%) and celery with the lowest yield (9.8%).

**Figure 1:**
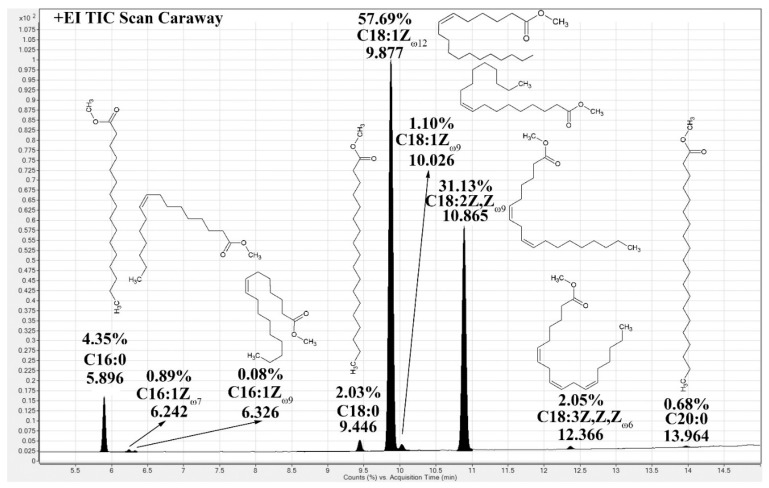
Electron ionization (EI) Total ion chromatogram of caraway FAMES.

**Figure 2:**
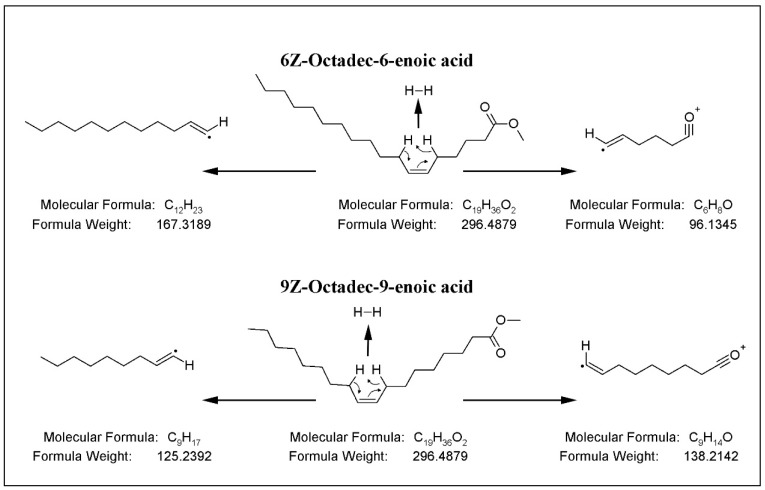
Proposed fragmentation pattern in the EI mode for differentiation between Z6 and Z9 18:1 fatty acids.

GCMS analysis of the fatty acids methyl esters (FAMES) of seeds oil was achieved with base line resolution for all samples as shown in Figure 1 for the caraway sample as a representative example of the analysis. The identification of petroselinic acid methyl ester was confirmed based on retention time, exact molecular weight (296.27008) and excellent fit with mass spectral library search. Petroselinic acid was differentiated from oleic acid by the presence of the radical cation of *m*/*z* = 96 corresponding to the cleavage of the double bond at the 6 position and loss of a hydrogen molecule. On the other hand, oleic acid 9*Z* 18:1 would produce a radical cation with *m*/*z* = 138 as shown in Figure 2. Duy *et al.* (2009) [13] analyzed fatty acids compositions for caraway, carrot and celery seeds using gas chromatography retention time analysis without conformation of structure by mass spectra and reported similar results but lack structural conformation.

**Table 1:** Relative percentage composition of FAMES in each of the nine *Apiaceae* oil samples.

Fatty Acids	Dill	Caraway	Cumin	Coriander	Anise	Carrot	Celery	Fennel	Khella
C16:0	5.79 ± 0.41	4.35 ± 0.51	4.67 ± 0.11	4.25 ± 0.10	4.75 ± 0.11	5.29 ± 0.15	8.51 ± 0.98	4.71 ± 0.12	4.92 ± 0.09
C16:1	0.52 ± 0.05	0.89 ± 0.09	0.33 ± 0.01	0.46 ± 0.05	0.29 ± 0.04	0.62 ± 0.09	0.38 ± 0.07	0.45 ± 0.05	0.26 ± 0.01
C16:1	nd *	0.08 ± 0.01	0.31 ± 0.01	0.26 ± 0.03	0.27 ± 0.05	0.64 ± 0.10	0.14 ± 0.01	0.08 ± 0.01	0.23 ± 0.01
C18:0	1.61 ± 0.08	2.03 ± 0.11	nd *	nd *	1.17 ± 0.25	0.91 ± 0.11	2.03 ± 0.04	1.23 ± 0.09	1.08 ± 0.11
6ZC18:1	79.91 ± 1.16	57.69 ± 0.91	61.83 ± 1.97	79.78 ± 2.61	66.26 ± 1.81	81.20 ± 2.41	65.79 ± 1.81	81.95 ± 2.41	74.95 ± 2.15
9ZC18:1	nd *	1.10 ± 0.05	1.40 ± 0.04	nd*	1.47	nd *	nd *	nd *	nd *
C18:2	10.80 ± 0.47	31.13 ± 0.81	30.44 ± 0.91	14.85 ± 0.31	24.58 ± 0.12	10.02 ± 0.11	21.65 ± 0.65	10.97 ± 0.31	16.68 ± 0.16
C18:3	0.70 ± 0.03	2.05 ± 0.05	0.61 ± 0.02	0.17 ± 0.03	0.75 ± 0.09	0.61 ± 0.05	1.01 ± 0.09	0.32 ± 0.01	1.41 ± 0.11
C20:0	0.52 ± 0.06	0.68 ± 0.04	0.15 ± 0.01	0.12 ± 0.02	nd *	0.32 ± 0.03	0.33 ± 0.07	0.22 ± 0.01	0.25 ± 0.01
% Yield ^§^	20.5	14.3	23.4	16.8	15.3	17.9	9.8	14.6	12.6

nd * is not detected. Yield ^§^ based on recovered neutral lipids.

### 2.2. Identification of Triacylglycerols TAG

**Figure 3:**
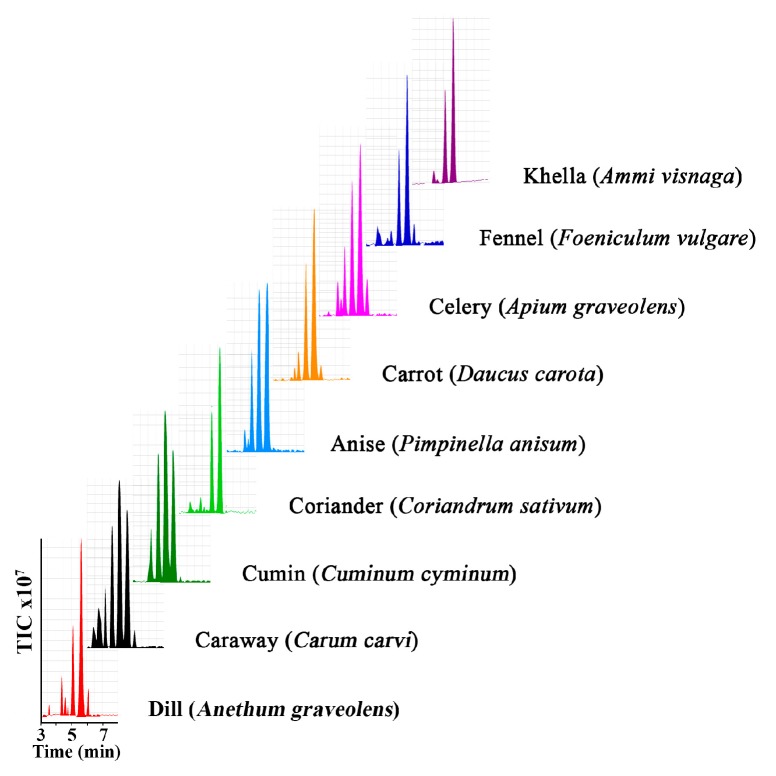
Total ion chromatogram of the nine examined samples.

**Figure 4:**
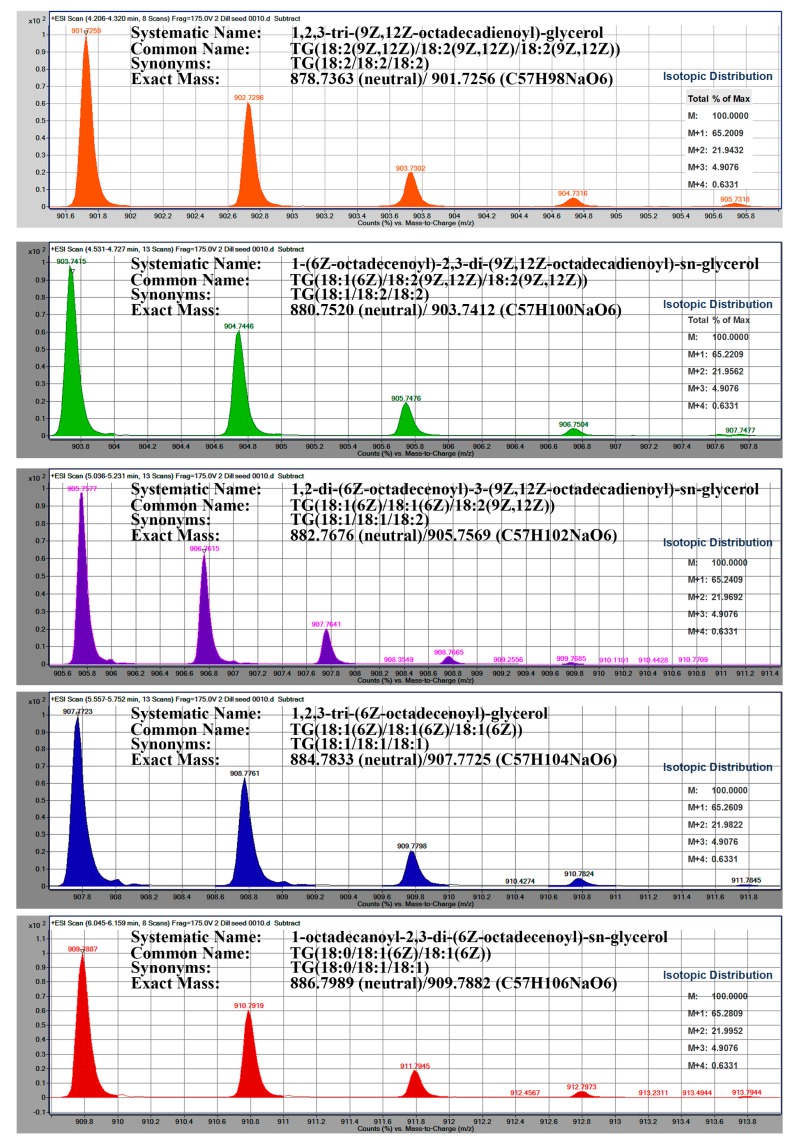
Natural isotope abundance for the five major TAG molecular ions.

TAG composition in the oil samples was invigilated using the accurate mass LC-QTOF system. Five major TAGs were found in all samples, however, were at different relative concentrations for each sample. Ions were identified as positive ion sodium adducts with exact masses of 901.7256, 903.7412, 905.7562, 907.7720 and 909.7887. Additional minor TAGs were also identified, but were not found in all samples and were not analyzed further. Although The ion [M + Na]^+^ with the accurate mass equivalent 901.7256 ± 0.0001 represents 186 possible TAG isomers, this number can be reduced to 16, by eliminating TAGs that have any fatty acid that was not detected in the oil (Table 1). Similarly, the second TAG has an accurate mass of 903.7412 representing a possible 405 isomers than be reduced to 24 isomers. The third TAG has an accurate mass of 905.7569 representing a possible 405 isomers than be reduced to 33 isomers. The fourth TAG has an accurate mass of 907.7725 representing a possible 342 isomers than be reduced to 35 isomers by eliminating fatty acids that were not detected by FAMES analysis. The fifth TAG has an accurate mass of 909.7882 representing a possible 291 isomers than be reduced to 33 isomers by eliminating fatty acids that were not detected by FAMES analysis. A list of all of the possible fatty acids combinations for the observed [M + Na]^+^ ions and their reduced numbers of isomers according to the identified fatty acids concluded by FAMES analysis is shown in Table 2. Total ion chromatogram of the nine examined samples are shown in Figure 3. Natural isotope abundance for the five major TAG molecular ions are shown in Figure 4. Percentage relative concentration of the detected major molecular ions in each sample is shown in Table 3. Minor identified TAGs and their distribution in the nine tested *Apiaceae* seeds are shown in Table 4.

**Table 2:** Possible numbers of structural isomers and region isomers of the predicted TAGs.

Accurate mass (Possible Isomers)	Suspected Isomers	Regioisomers
901.7256 (186 isomers)	18:1Z9/18:2/18:3	6
	18:1Z6/18:2/18:3	6
	18:0/18:3/18:3	3
	18:2/18:2/18:2	1
	**Total**	**16**
903.7412 (405 isomers)	18:1Z9/18:2/18:2	3
	18:1Z6/18:2/18:2	3
	18:0/18:2/18:3	6
	18:1Z9/18:1Z9/18:3	3
	18:1Z6/18:1Z6/18:3	3
	18:1Z9/18:1Z6/18:3	6
	**Total**	**24**
905.7569 (405)	18:0/18:1Z9/18:3	6
	18:0/18:1Z6/18:3	6
	16:1/18:3/20:0	6
	18:0/18:2/18:2	3
	18:1Z6/18:1Z6/18:2	3
	18:1Z9/18:1Z9/18:2	3
	18:1Z9/18:1Z6/18:2	6
	**Total**	**33**
907.7725 (342)	18:0/18:0/18:3	3
	18:1Z6/18:1Z6/18:1Z6	1
	18:1Z9/18:1Z9/18:1Z9	1
	18:1Z9/18:1Z9/18:1Z6	3
	18:1Z9/18:1Z6/18:1Z6	3
	18:0/18:1Z9/18:2	6
	18:0/18:1Z6/18:2	6
	16:0/18:3/20:0	6
	16:1/18:2/20:0	6
Total	**Total**	**35**
909.7882 (291)	18:0/18:1 Z9/18:1Z9	3
	18:0/18:1 Z6/18:1Z6	3
	18:0/18:1 Z9/18:1Z6	6
	18:0:18:0/18:2	3
	16:0:18:2/20:0	6
	16:1:18:1Z9/20:0	6
	16:1:18:1Z6/20:0	6
	**Total**	**33**

**Table 3:** Percentage relative concentration of the detected major molecular ions in each sample.

Identified TAGs	Dill	Caraway	Cumin	Coriander	Anise	Carrot	Celery	Fennel	Khella
TG18:2/18:2/18:2 C_57_H_98_O_6_Na		6.17	7.78	0.68	1.25	1.85	2.01	nd *	nd *
901.7256									
4.20 min									
TG18:1/18:2/18:2 C_57_H_100_O_6_Na	3.31	16.24	20.15				10.16	2.76	
903.7412									
4.63 min									
TG18:0/18:3/18:2 C_57_H_100_O_6_Na				0.87	12.75	5.27			0.86
903.7412									
4.63 min									
TG18:1/18:1/18:2 C_57_H_102_O_6_Na		29.71	37.88			25.99			
905.7569									
5.13 min									
TG18:1/18:2/18:1 C_57_H_102_O_6_Na	22.36			28.38	25.41		24.38	17.68	26.33
905.7569									
5.13 min									
TG18:1/18:1/18:1 C_57_H_104_O_6_Na	58.40	24.73	25.34	62.72	31.22	49.25	37.17	40.04	51.95
907.7725									
5.66 min									
TG18:0/18:1/18:1 C_57_H_106_O_6_Na		3.27	0.63	0.32		2.64	5.34	3.71	nd *
909.7882									
TG18:1/18:0/18:1 C_57_H_106_O_6_Na	5.06				0.79				nd *
909.7882									
6.50 min									

nd * is not detected.

**Table 4:** Minor identified TAGs and their distribution in the nine tested *Apiaceae* seeds.

TAGs	Dill	Caraway	Cumin	Coriander	Anise	Carrot	Celery	Fennel	Khella
TG16:0/16:0/16:0 C_51_H_98_O_6_Na	x	x	x	x	x		x		x
829.7256									
4.20 min									
TG16:0/18:2/18:2 C_55_H_98_O_6_Na		x				x	x		x
877.7248									
4.73 min									
TG16:1/18:1/18:2 C_55_H_98_O_6_Na						x			x
877.7248									
4.73 min									
TG16:1/18:0/18:3 C_55_H_98_O_6_Na	x						x		
877.7248									
4.73 min									
TG18:3/18:3/18:3 C_57_H_92_O_6_Na			x		x	x	x		
895.6786									
3.73 min									
TG16:1/18:0/20:0 C_57_H_108_O_6_Na	x								
911.8038									
6.50 min									
TG18:0/18:0/18:1 C_57_H_108_O_6_Na					x				
911.8038									
6.50 min									
TG18:0/18:0/18:0 C_57_H_110_O_6_Na		x			x		x		
913.8195									
7.12 min									
TG18:1/18:1/20:0 C_59_H_110_O_6_Na	x	x	x	x	x	x	x	x	x
937.8186									
6.60 min									
TG18:2/20:0/20:0 C_61_H_114_O_6_Na	x	x	x	x	x	x	x		x
965.8508									
6.73 min									

### 2.3. Determination of Regioisomers by MS/MS Experiments

There are large number of different triacylglycerols species (TAGs), which differ in the total length of acyl chains and their degree of unsaturation. Isobaric TAGs are designated by having the same number of carbon atoms and double bonds possessing acyl chains with different length, position and configuration of double bonds. The acyl chain may be located in three different positions of TAG, known as the sn-1, sn-2, or sn-3 positions. The regiospecific analysis is restricted to determine the acyl chains orientation as sn-1(3) and sn-2 positions. Mass spectral fragmentation can provide information about the stereochemistry and regiospecific identification of the TAGs [14,15,16,17,18].

Based on our finding on the fragmentation of standard authentic TAGs and available published work [19,20], it can be concluded that fragmentation pattern for TAGs follow the following scheme: The most abundant ions observed in the mass spectra of TAG are positive ions containing two complete fatty acid chains and those containing only one complete chain corresponding to the loss of one or two fatty acids respectively. Two ions were found corresponding to the ion remaining after the loss of one acyloxy group from the molecular ion [M-RCOO]^+^, and the other corresponding [M-RCOOH]^+^. If the three fatty acids are different, there would be three different [M-RCOO]^+^ and [M-RCOOH]^+^ ions, corresponding to the loss of each different acyloxy group. However, due to steric and electronic property, the loss of the fatty acid located in the sn2 is energetically less favorable than one at the end positions (sn1 and sn3) [21,22]. Therefore, ions corresponding from the loss of fatty acids from the sn1 and sn3 positions are much more abundance the ion corresponding to the loss of fatty acid at the sn2 position [21,22]. The other class of intense ions characteristic of the individual fatty acid chains is that group of ions containing only one of the fatty acid moieties. The simplest of these are the acyl ions, RCO^+^. Since each fatty acid chain produces an RCO^+^ ion, if two or three of the chains are different, two or three different RCO^+^ are found but more for the sn1 and sn3 positions. Another ion of relatively high intensity is that corresponding to the acyl ion plus 74 amu equivalent to C_3_H_6_O_2_ corresponds to the glyceryl moiety [RCO+74] + ion.

Applying the experimental results observed with authentic TAGs and the above reported information about fragmentation of TAGs to the five major TAGs that were identified in the nine fruits of the Apiaceae family. Two of the identified TAG were found to have the same fatty acid in all of the three positions (1, 2 and 3) with an accurate mass of 901.7256 (C_57_H_98_O_6_Na) and 907.7725 (C_57_H_104_O_6_Na) corresponding to TG18:2/18:2/18:2 and TG18:1/18:1/18:1, respectively, for the TAG. This was also confirmed by the presence of the ion at *m*/*z* of 621.4765 corresponding to loss of the fatty acid 18:2 as [M-RCOO]^+^ and a minor ion at 620.4639 as [M-RCOOH]. Ion at 337.2602 corresponding to ion of RCO+74 for the 18:2 fatty acid and the ion 263.2367 corresponding to the RCO+ for the fatty acid 18:2 confirming the structure of TAG as trilinoleate (LLL), which was also confirmed by the HPLC retention time compared to the authentic sample. With regard to the second symmetrical TG18:1/18:1/18:1 having an accurate mass of 907.7725 (C_57_H_104_O_6_Na), it was also found to be consistent with the proposed fragmentation by showing a single ion of *m*/*z* = 625.5168 corresponding to loss of the fatty acid 18:1 as [M-RCOO]^+^. Ion at *m*/*z* of 339.2888 corresponding to ion of RCO+74 for the 18:1 fatty acid and the ion 265.2526 corresponding to the RCO^+^ for the fatty acid 18:1 confirming the structure of TAG as tripetroselinate (PsPsPs), which was also confirmed by the HPLC retention time compared to the authentic sample. The structure may contain some of the oleic acid that cannot be differentiated from petroselinic acid with the available mass spectral data except that oleic acid with very minor concentration (FAMES analysis) compared to petroselinic acid, which was found to be the most abundant fatty acid in all samples. Both TAG (901 and 907) were identical in all of the nine analyzed samples and they only differ in their relative concentration.

For the other multiacyl TAGs, we found that the ion with the accurate mass of 903.7412 fragmented to give two major ions with *m*/*z* = 621.48327 and 623.40732 corresponding to loss of the fatty acid 18:1 as [M-RCOO]^+^ and the loss of the fatty acid 18:2 as [M-RCOO]^+^, respectively, indicating that the two fatty acids are most probably occupy the sn1 (3) positions. The third fatty acid located at the sn2 position must be an 18:2 based on the presence of the ion at 337.2678 corresponding to ion of RCO+74 for the 18:2 fatty acid and the ion 263.23363 corresponding to the RCO^+^ for the fatty acid 18:2 confirming the structure as TG18:1/18:2/18:2. For the other multiacyl TAGs we found that the ion with the accurate mass of 905.7569 [C_57_H_102_O_6_Na]^+^ Fragmented to give two major ions with *m*/*z* = 623.48327 and 625.51296 corresponding to loss of the fatty acid 18:1 as [M-RCOO]^+^ and the loss of the fatty acid 18:2 as [M-RCOO]^+^, respectively, indicating that the two fatty acids are most probably occupy the sn1(3) positions. The third fatty acid located at the sn2 position must be an 18:1 based the presence of the ion at 339.2678 corresponding to ion of RCO+74 for the 18:1 fatty acid and the ion 265.23363 corresponding to the RCO^+^ for the fatty acid 18:1 confirming the structure as TG18:1/18:1/18:2. Fragmentation pattern also showed that this triglyceride may contain a smaller amount of another regioisomer having the same exact mass with structure corresponding to TG18:1/18:2/18:1.

**Figure 5:**
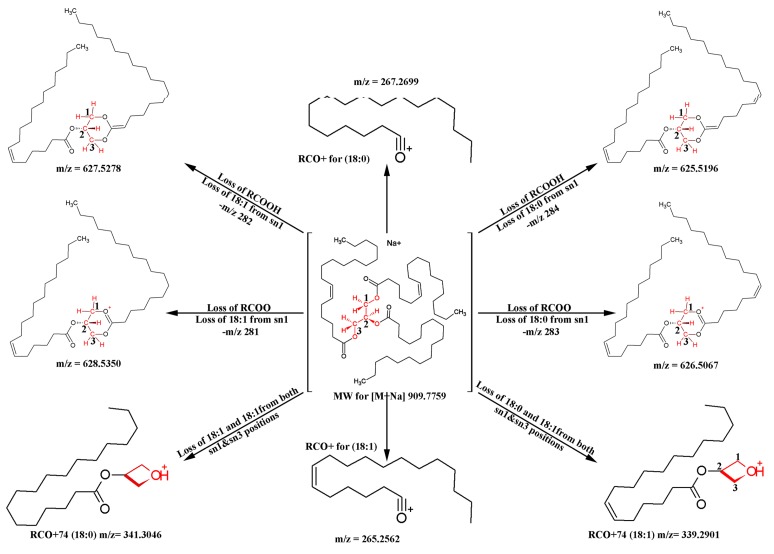
Electrospray Ionization (ESI) positive fragmentation pattern of the TAG 909.7759 molecular ion according to the MS/MS experiments.

For the other multiacyl TAGs we found that the ion with the accurate mass of 909.7882 [C_57_H_106_O_6_Na]^+^ fragmented to give two major ions with *m*/*z* = 625.5137 and 627.6274 corresponding to loss of the fatty acid18:0 as [M-RCOO]^+^ and the loss of the fatty acid 18:1 as [M-RCOO]^+^ respectively, indicating that the two fatty acids are most probably occupy the sn1(3) positions. The third fatty acid located at the sn2 position must be an 18:0 based the presence of the ion at 341.8040 corresponding to ion of RCO + 74 for the 18:0 fatty acid and the ion 267.2650 corresponding to the RCO^+^ for the fatty acid 18:0 confirming the structure as TG18:1/18:0/18:1. Fragmentation pattern also showed that this triglyceride may contain a smaller amount of another regioisomer having the same exact mass with structure corresponding to TG18:0/18:1/18:1. The fragmentation pattern and ions identifications are shown in Figure 5.

## 3. Experimental Section

### 3.1. Materials

All general use solvents and chemical reagents were purchased from VWR (Sugar Land, TX, USA). LCMS water and solvents were purchased from J.T.Baker (Sugar Land, TX, USA). Isooctane and 1% Boron trifluoride in methanol were obtained from Sigma-Aldrich (Milwaukee, WI, USA). Glyceryl trilinoleate (LLL), glyceryl trilinolenate (LnLnLn), glyceryl trioleate (OOO), glyceryl tripalmitate (PPP), glyceryl tripetroselinate (PsPsPs), and glyceryl tristearate (SSS) TAG were obtained from Sigma-Aldrich (USA) and used as standards for LC retention time. Complete set of fatty acids methyl esters both saturated and unsaturated from C4 to C26 were obtained from Sigma-Aldrich (USA) and were used for GC retention time comparison.

### 3.2. Oil extraction

Mature dry seeds of the nine selected *Apiaceae* species were obtained from the seed company KATO Aromatic of Egypt. The seeds (100 g) were finely ground in an electric grinder. The oil from the resulting flour was extracted in glass percolators in 500 mL of chloroform/methanol (2:1 *v*/*v*) at room temperature. The extract was dried over anhydrous sodium sulfate, filtered and concentrated at less than 50 °C in a rotary evaporator, yielding yellow to greenish oils, which were kept sealed under argon at −20 °C until further use. The crude lipid extract was purified to obtain neutral glycerides using solid phase extraction on Strata NH2 cartridge (Strata C18E, 500 mg/3 mL (P/N 8B-S001-HBJ, Phenomenex, Torrance, CA, USA). Starta C18E cartridge was conditioned using 500 µL acetonitrile and equilibrated by 500 µL of water before applying the crude extract (1 mL in chloroform methanol) cartridge was then washed with 5% methanol in water, dried for 1 min and eluted with acetonitrile. Second eluent was passed into a NH_2_ Sorbent cartridge, which was previously conditioned using hexane, and neutral lipids were eluted with 4 mL of Chloroform/2-propanol (2:1 *v*/*v*).

### 3.3. Fatty Acids Methyl Esters (FAMES) Preparation Analysis

Ten microliters of the neat neutral lipid extract was added to 2 mL of 1% BF_3_ in methanol, mixed by vortex and placed on the heating block at 75 °C for 1 h. After cooling down to room temperature, 1 mL of saturated sodium chloride solution was added to step the reaction and FAMES were extracted in 2 mL of iso-octane, passed through anhydrous sodium sulfate and transferred to GC auto- sampler vials.

### 3.4. Accurate Mass GC-QTOF

Agilent 7200 accurate mass GC-QTOF System was used for analyzing FAMES samples. GC separation was done using a ZB-Wax plus column 30 m × 0.25 mm, 0.25 µm film thickness column (Phenomenex, Torrance, CA, USA) run under average velocity of 1 cm/sec with a hold up time of 1 min. Temperature programming started at 175 °C, held for 3 min, and heated up to 225 °C at a rate of 2.5 °C/min, and held at 225 °C for 7 min. Total run time was 30 min; solvent delay 2 min; and equilibrium time 2 min. Nitrogen collision gas 1.5 mL/min; mass range from 100 to 400 *m*/*z*; quadrupole temperature at 150 °C injected volume 1 µL; split mode 50:1. The measurements and post-run analyses were controlled by Mass Hunter Qualitative Analysis B.06.01 (Agilent Technologies). FAMES were identified based on their retention times and their accurate mass data. Their electron ionization fragmentation and mass spectral data were also searched using Wiley10NIST mass spectral database.

GCMS analysis of the fatty acids methyl esters (FAMES) of seeds oil was achieved with base line resolution for all samples, as shown in Figure 1 for the caraway sample as a representative example of the analysis. The identification of petroselinic acid methyl ester was confirmed based on retention time, exact molecular weight (296.27008) and excellent fit with mass spectral library search. Petroselinic acid was differentiated from oleic acid by the presence of the radical cation of *m*/*z* = 96 corresponding to the cleavage of the double bond at the 6 position and loss of a hydrogen molecule. On the other hand, oleic acid z9 18:1 would produce a radical cation with *m*/*z* = 138, as shown in Figure 2. Duy *et al.* (2009) [13] analyzed fatty acids compositions for caraway, carrot and celery seeds using gas chromatography retention time analysis without conformation of structure by mass spectra and reported similar results but lack structural conformation*.*

### 3.5. LC-QTOF

Agilent 6530 accurate mass LC-QTOF system equipped with 1290 binary pump, Phenomenex Kinetex C18 100 mm × 3.0 mm 2.6 µm column. Solvents A was made of isopropanol; B1 acetonitrile run in a gradient protocol starting at 50% A, 50% B with a flow 0.2 mL/min reaching 100% solvent A at 10.0 min from the start, and held at 100% A for additional 5 min. Injection volume was 0.2 µL; mass range from 100 *m*/*z* to 1500 *m*/*z*, with a total run time of 15 min. The LC-QTOF instrument was operated under the following conditions: Ion source ESI + Agilent Jet Stream Technology in positive ionization mode. The Jet Stream ESI source was operated in negative mode, and instrument parameters were set as follows: sheath gas temperature, 350 °C; sheath gas flow, 8 L/min; nebulizer, 20 psi; dry gas temperature, 300 °C; dry gas flow, 5 L/min; and capillary entrance Voltage, 3500 V. Fragmentor and Skimmer1 were operated at 190 and 65 V, respectively. The MS scan data were collected at a rate of 1.02 spectra/s in the range of *m*/*z* 100–2000. All the MS data were collected with Mass Hunter Data Acquisition B.06.00 (Agilent Technologies), and Mass Hunter Qualitative Analysis B.06.00 (Agilent Technologies) was applied to identify lipid species. All EICs were obtained with ±10 ppm *m*/*z* expansion. Mass Profiler Professional 2.1 (Agilent Technologies) and Microsoft Office Excel 2013 (Microsoft, Redmond, WA, USA) were used for statistical data analysis and data visualization.

### 3.6. LC-QTOF MS/MS Experiment

MS/MS method development gave energies of 50 eV, run time 15 min; ion source: positive; source gas temperature 300 °C; drying gas 3.0 L/min; nebulizer 15 psig; sheath gas temperature 125 °C; sheath gas flow 3.0 L/min; dual AJS ESI; V cap 4000 V; capillary 0.063 µA; nozzle Voltage 0 V; chamber 5.33 µA; ms/ms mass range 100 *m*/*z* to 1600 *m*/*z*; acquisition rate 1 spectrum/s; acquisition time 1000 ms/spectrum; 13,503 transients/spectrum; auto recalibration reference mass window, detection window 100 ppm; minimum height 1000 counts; polarity type: positive; offset: 15; ms/ms absolute threshold 5; reference threshold 0.01%.

## 4. Conclusions

We report here for the first time the use of both accurate mass gas chromatography and liquid chromatography quadrupole time of flight mass spectrometry analysis for the identification of the composition and regiodistribution of triacylglycerols in nine of the most used *Apiaceae* seed oils. It also confirms the fatty acids composition of these seed oils, in addition to determining the presence of minor 16:1 and 18:1 positional isomers. The results show that the TAG composition and the positional distribution of petroselinic acid on the TAG of the examined *Apiaceae* seed oils were similar but different in their relative concentration. Among the species studied in this work, fennel seed oil contained the highest levels of petroselinic acid, making it potentially interesting source of petroselinic acid.
